# Psychological well-being in people with multiple sclerosis: a descriptive review of the effects obtained with mindfulness interventions

**DOI:** 10.1007/s10072-021-05686-1

**Published:** 2021-10-25

**Authors:** Marcella Di Cara, Denise Grezzo, Rosanna Palmeri, Viviana Lo Buono, Emanuele Cartella, Katia Micchia, Caterina Formica, Carmela Rifici, Edoardo Sessa, Giangaetano D’Aleo, Giuseppa Maresca, Placido Bramanti, Francesco Corallo

**Affiliations:** IRCCS Centro Neurolesi “Bonino-Pulejo”, S.S, 113 Via Palermo, C.da Casazza, 98124 Messina, Italy

**Keywords:** Multiple sclerosis, Mindfulness, Psychotherapy, Anxiety, Depression, Pain

## Abstract

Multiple sclerosis is a neuroinflammatory and neurodegenerative disease causing several psychosocial problems that significantly impairs quality of life. The most common physical and mental symptoms are anxiety, depression, stress, fatigue, and pain. Several studies investigated the effectiveness of non-pharmacological approaches in improving psychological well-being. This review focused on the impact of mindfulness interventions in patients with multiple sclerosis to reduce psychopathological symptoms and improve well-being. We searched on PubMed database and screening references of included studies and review articles for additional citations. From initial 107 studies, only 8 met search criteria. Our studies showed the efficacy of mindfulness treatment with a reduction in depressive symptoms, a better quality of life (both mental and physical), and a decreased level of fatigue. Findings demonstrated that mindfulness is useful for the improvement of psychological symptoms and pain management and this improvement has also been shown to have a positive impact on the quality of life and coping and adaptation strategies. However, according to the poor available clinics evidence, on cannot conclude that mindfulness interventions are superior to other active interventions in the treatment of psychological symptoms of SM.

## Introduction

Multiple sclerosis (MS) is a neuroinflammatory and neurodegenerative disease characterized by an unpredictable development that can be relapsing or progressive. Its estimated incidence is over 2.5 million people worldwide, classifying it as a major cause of non-traumatic disability in young adults in many countries (Browne et al., 2014).

People with MS have several psychosocial problems because of the disease that significantly impair their quality of life (Di Cara et al., 2020).

The most common physical and mental symptoms are anxiety, depression, stress, fatigue, and pain. The study by Nauta et al. [21] shows that stressful life events for people with MS significantly worsen neurological symptoms and quality of life.

For this reason, many studies in recent years have focused on approaches that can improve psychological well-being [11].

Several studies have shown, for example, that psychopharmacological treatments using selective serotonin, although effective in treating depression, have several side effects and a high dropout level [8, 12]. On the other hand, it would seem that psychological counselling and psychotherapeutic treatments, in particular cognitive behavioral therapy (CBT), have a significant effect on depression, even in the long term [33].

Mindfulness interventions have been shown to be useful for the improvement of psychological symptoms and pain management [1]. This improvement has also been shown to have a positive impact on quality of life and coping and adaptation strategies [7, 20].

Mindfulness is a technique that involves awareness-oriented meditation with attention to the present moment. This involves the recognition of thoughts, emotions, and sensory experiences, using a non-judgmental attitude of openness and receptivity (Didonna F.et al., 2009; Chiesa A. et al., 2010; Kabat-Zinn J.et al., 2003). During the last 10 years, a strong interest has developed in the empirical investigation of the application of mindfulness (Chiesa et al., 2013) as a possibility to deal with various psychological and physical disorders [4, 18]. This technique has been shown to be effective in reducing stress and symptoms caused by different pathologies including chronic pain, fibromyalgia, psoriasis, depressive, and eating disorders (Keune, 2010,Rosenzweig 2010).

This descriptive review focused on literature studies that investigated the impact of mindfulness interventions in patients with MS on reduce psychopathological symptoms and improve well-being.

## Methods

### Search strategy

**Fig. 1:**
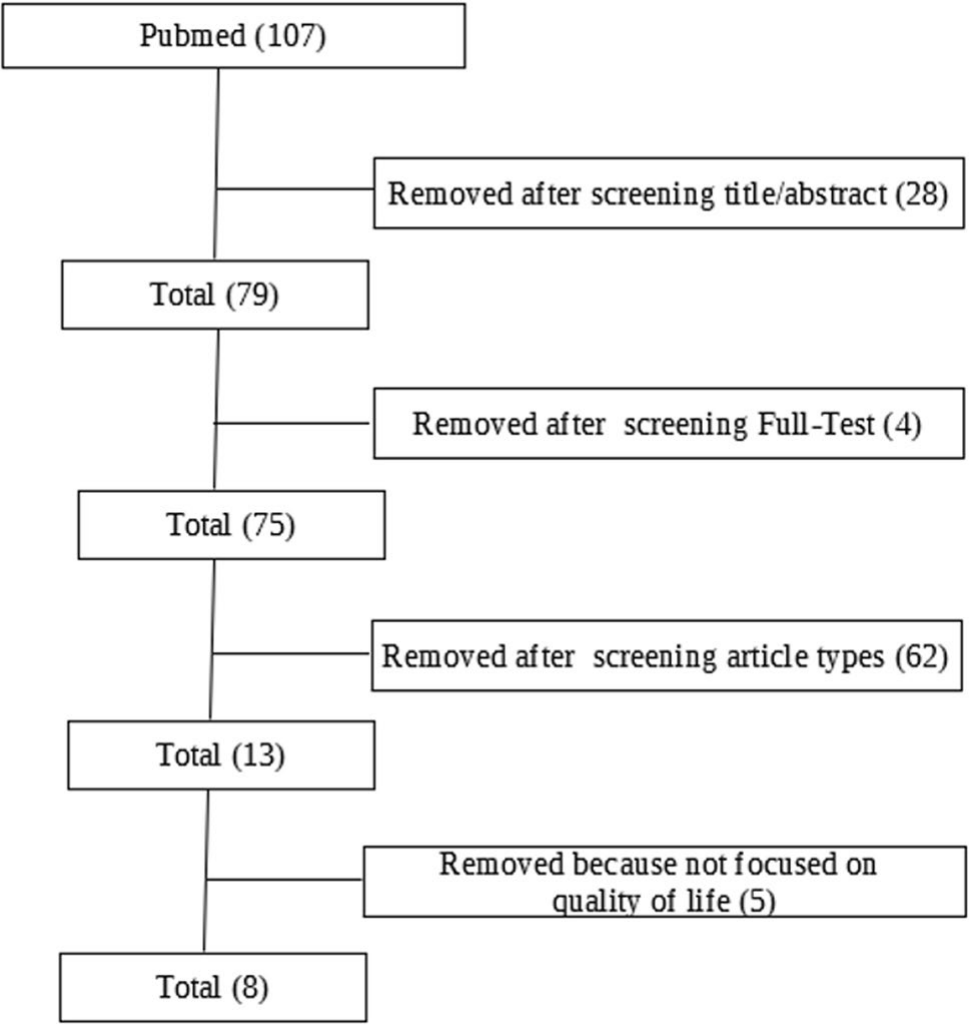
Search and selection of eligible articles

The studies have been selected from the PubMed database (2016, year of the first article selected — 2019) (Fig. 1).

The survey combined the terms “mindfulness” (All Fields) and “Multiple Sclerosis” (All Fields).

The research terms were identified in the title and abstract, and only studies published in English were considered.

After examining the selected studies by removing those that differed from the criteria, we selected due to the characteristics of the methodologies used, the articles were evaluated according to the title, the abstract, and the research methodologies used.

The selection of studies was related to the study design, participants, interventions, and outcomes.

Specifically, studies were selected according to the following criteria:

1. Studies already completed at the time of the research;
2. Sample composed of patients with MS;
3. Studies that assessed the psychological well-being of patients;
4. Studies which deepen mindfulness-based intervention;
5. Case studies were excluded.

## Result

Only 8 studies met the inclusion criteria. The number of subjects involved in the research was 416 MS patients (301 received treatment using mindfulness techniques, 35 were part of a psychoeducational group, and 55 received no treatment).

All patients included in the different research designs were affected by MS and followed by several outpatient clinics in Italy and abroad. Participation in the study was on a voluntary basis and assignment to the experimental or random control group. None of the participants had a history of psychiatric disorders. The samples were made up of subjects belonging to different sexes distributed randomly, except for one study that focused on a sample of women only [19]. The selected subjects were all over the age of majority and there is no indication as to the qualification or onset of the disease.

The aims and results for each study are summarized in Table 1.

**Table 1:** Aims and results for each study

Authors, published	Aim	Sample (*n*)	Test to evaluate psychological function	Neuropsychological Evaluation	Results
Roos J. Blankespoor et al	Investigate the effectiveness of mindfulness-based stress reduction (MBSR) on the stress and cognitive functions of MS patients	31 mindfulness group	BDI MSQL-54 CIS-F FFMQ SCS	MACFIMS MMQ Rey Auditory Il test Misure di LLT PASAT Letter-number sequencing test	After participating in MBSR, patients experienced less depressive symptoms, improved quality of life, both in the physical and mental domain, and patients were less fatigued, albeit minimal, changes in cognitive functioning were reported with reference to memory
Robert Simpson et al	Test the feasibility and probable efficacy of a standard MBSR course for people with MS	25 mindfulness group 25 control group	Perceived Stress Scale-10EQ-5D-5 L MSQLI Mindful Attention Awareness Scale — MAAS) SCS ELQ		Recruitment, retention, and data collection demonstrate that a RCT of MBSR is feasible for people with MS
Sara Carletto et al	Evaluate the effectiveness of an affective body based on the awareness intervention group by comparing it with a psychoeducational intervention, by means of a randomized controlled clinical trial	36 mindfulness group 35 psycho-educational group	FSS BDI-II BAI PSS B-IPQ FAMS M.I.N.I.-Plus		The awareness intervention improved the quality of life of the patients and the perception of the disease; these improvements were maintained at the follow-up evaluation
Rachel M. Gilbertson et al	This study examined the feasibility of using mindfulness in motion in people with multiple sclerosis (MS) and the effect of this program on stress, anxiety, depression, fatigue, and quality of life in people with MS	22 mindfulness group	MHI MFIS FFMQ SF-36		Mindfulness in motion proved to be a feasible program yielding positive results, supporting the need for research to determine the extent to which the program can improve quality-of-life outcomes for people with MS
Angela Senders et al	The objective of this study was to evaluate the association between pain interference and trait mindfulness in people with MS	132 mindfulness group	Patient-Reported Outcomes Measurement Information System (PROMIS) FFMQ		These results suggest a clinically significant association between mindfulness and pain interference in MS and support further exploration of mindfulness-based interventions in the management of MS-related pain
Cristiano Crescentini et al	To evaluate the effects of an 8-week mindfulness-oriented meditation training on the personality profiles, anxiety and depression symptoms, and mindfulness skills of a group of patients with MS	17 mindfulness group 16 control group	TCI BFI STAI BDI FFMQ		The data support the utility for patients with MS of therapeutic interventions based on mindfulness meditation that may lead to enhanced character and self-maturity
Somayeh Nejati et al	To evaluate effect of group mindfulness-based stress reduction and consciousness yoga program on quality of life and fatigue severity in patients with MS	12 mindfulness and yoga group 12 control group	SCID-I/CV FSS MSQOL-54		The results show that the program is effective in reduction of fatigue severity and improving some subscales of quality of life in MS patients
Bentolhoda Kolahkaj et al	To evaluate effect of mindfulness-based stress reduction (MBSR) therapy on quality of life in women with multiple sclerosis	26 mindfulness group 27 control group	SCID-I QOL		In the MBSR group, the mean subscales of QOL had more significant reduction compare to control group. Also the improvement of all subscales of mental and physical QOL continued after 2 months later in follow up stage The findings suggest that MBSR is useful for improving the quality of life in patients with MS

### Efficacy of mindfulness treatment

Anxiety, depression, psychological distress, and fatigue are common comorbidity (Goric et al., 2021) among people with MS, with estimation prevalence variable and largely dependent on the variable measurement tool used in the different studies [32]. These symptoms can have a devastating impact on daily functioning and well-being and are associated with a worse prognosis [14]. Many data in the literature have focused on cognitive and physical disability resulting from MS. However, only in recent years, some authors have evaluated the effectiveness of mindfulness, as alternative treatment or in addition to drug therapy, in reducing psychiatric symptoms and improving the quality of life in MS patients. Results of these studies have indicated mindfulness interventions are a promising choice in treating psychological functioning in MS patients, e.g., in psychological distress reduction,nevertheless, literature data are discordant regarding temporal duration of the effects.

In the study by Simpson et al. [31], the perceived stress levels, in MS patients, had decreased significantly immediately after the mindfulness sessions but at the 3-month follow-up, they had not remained as low. In addition, the quality of life values had not changed significantly either immediately or 3 months after the end of the mindfulness sessions. Although the short-term anxiety-depressive symptomatology had achieved good results with a reduction in symptoms, in the long term, what reported significant results were the acquisition of greater awareness, self-confidence. On contrary, in the study conducted in Iran by Kolahkaj et al. [19], the effectiveness of mindfulness treatment on stress reduction in women with MS to improve their quality of life remained constant over time as assessed in the follow-up sessions.

Other authors have more specifically investigated the effect of mindfulness on psychiatric symptoms in MS patients.

Blankespoor et al. [26] used a mental training program developed in 8 sessions to evaluate the effect of mindfulness treatment on psychological functioning and quality of life, and increase the cognitive functioning of MS patients.

The results obtained by this research group suggested that following the mindfulness sessions, patients experienced a reduction in depressive symptoms, a better quality of life (both mental and physical), and a lower level of fatigue than before starting the sessions. They also showed that after the sessions, the patients were more tolerant with themselves and able not to identify with negative thoughts and emotions all the time. Mindfulness, indeed, was effective in improving the way we observe and describe emotional experiences by learning to control reactions to them.

Also Crescentini et al. evaluated the influence of mindfulness on depressive symptoms and personality profiles, taking into account interventions oriented towards awareness on character scales (self-directionality, cooperativity, and self-transcendence) and on personality traits (consciousness, neuroticism, extraversion, pleasantness, and openness). The authors found increased awareness and decreased anxiety-depressive symptoms after the meditation training were observed. In line with this study was the results showed by Carletto et al. [3] that used mindfulness interventions aimed at increasing body awareness in the treatment of depression and improving the quality of life of caregivers was observed. This intervention included awareness sessions with accurate body scanning, breathing and walking mediation, yoga exercises, awareness in relational practices, and sensomotor psychotherapy. There were 8 weekly sessions that included a 7-h session. The control sample included a psychoeducational intervention. Mindfulness interventions were more effective than psychoeducation in reducing depressive symptoms.

Rachel et al. [25] using mindfulness in motion that include yoga movement, mental meditation, and relaxing music showed a decrease in psychiatric symptoms with significant changes in both physical and mental health in MS patients.

Conscious yoga program and mindfulness were used in the study by Nejati et al. [22] to evaluate the effectiveness of this treatment on stress reduction. Like other studies previously mentioned, a significant reduction of fatigue resulting in an improved the physical and mental quality of life and well-being of people with MS.

Only one study [30] assessed the effect of mindfulness interventions on chronic pain, a common symptom in people with MS. The results of the research showed that greater awareness of one’s body corresponds to lower levels of pain, shifting attention away from it. This suggests that interventions aimed at building awareness can improve trait awareness in people with different degrees of physical ability.

## Discussion

MS is a chronic autoimmune demyelinating disorder that affects the central nervous system and damages the myelin sheaths around the nerves, causing inflammation, loss of myelin, and axonal destruction. Clinical manifestations in MS vary according to the area of the central nervous system affected by demyelination and include sensorimotor, cerebellar, psychiatric, and cognitive alterations. Understanding the influence of these symptoms in a patient’s quality of life and their multifactorial nature seems to be fundamental to the development of new psychosocial interventions.

Mindfulness is a process that helps to develop a mental awareness oriented to the present of one’s feelings or body states [2], and it represents important treatment in management physical pain or suffering through meditation. Numerous literature date reports a great effectiveness of mindfulness interventions on reduce migraine pain both adult than children [28, 29], probably for effects on reduction concentration of biomarkers of inflammation [10]. Chronic pain is one of the most disabling symptoms in multiple sclerosis [24], along with anxiety and depression.

The studies reviewed suggested that mindfulness improves psychiatric symptoms regardless of severity, even when these symptoms are associated with other medical conditions. An only study focused on MS pain showing a strong and significant association between mindfulness and lower levels of pain interference.

It is possible that the effect of mindfulness on reduce SM symptoms is due to better stress management, since the patients begin to relate differently to their physical symptoms and are better prepared to cope with them. Howells et al. [13] have, indeed, shown that awareness-based interventions increase the capacity for attention and reduce the interference of irrelevant information. Mindfulness intervention seems to promote positive management mechanisms based on active problem-solving strategies.

A small number of works were included in this review since only six studies met the inclusion criteria. A meta-analysis was unable to be performed because quantitative information was not reported in the included studies.

In addition, excepting for Simpson et al. [31] and Carletto et al. [3], which adopted a follow-up respectively after 3 and 6 months, confirming the well-being maintenance after mindfulness intervention, the absence of a long-term follow-up examination (more than 6 months) makes it difficult to establish stability of the psychological changes observed. It is known that mindfulness interventions have a low attrition rate and no side effects, generally, have well received regardless of the specific subtype. However, it was difficult for some studies to definitively attribute the observed changes to the practice of mindfulness.

All studies, indeed, were based on self-report; therefore, the negative mood of the person with MS may have affected their responses and their perception of well-being after the interventions. Only Carletto et al. [3] involved caregivers, but the low number of participants was unable to evaluate the effects of the interventions on this population. Further investigations could consider the role of the caregiver both as an observer in change and in assessing whether caregivers would benefit from the treatment.

Mindfulness allows to intervene in a complete way on the person, focusing both on the physical and mental aspect, promoting a better knowledge of their body. Therefore, through a path of greater awareness seems to be possible to effectively manage symptoms of anxiety-depressive or physical fatigue. Mindfulness is well known to have positive effects on mental health among different clinical populations; however, recommendations cannot be made based on current evidence due to limited research and inadequate methodological rigor of published literature [35–37]. The mechanisms of action in these interventions that lead to beneficial physical and psychological outcomes have yet to be clearly identified and there is a lack of methodological rigor in the field of testing mechanisms of action which precludes definitive conclusions.

From a clinical standpoint, according to the poor available evidence, we cannot conclude that mindfulness interventions are superior to other active interventions in ameliorating all the considered outcomes, suggesting a role as complementation and not as replacement of the treatment of psychological symptoms in SM.
